# Facile Microemulsion Synthesis of Vanadium-Doped ZnO Nanoparticles to Analyze the Compositional, Optical, and Electronic Properties

**DOI:** 10.3390/ma12050821

**Published:** 2019-03-11

**Authors:** H. S. Ali, Ali S. Alghamdi, G. Murtaza, H. S. Arif, Wasim Naeem, G. Farid, Sadia Sharif, Muhammad Gul Bahar Ashiq, Syeda Ammara Shabbir

**Affiliations:** 1 Centre for Advanced Studies in Physics, GC University, Lahore 54000, Pakistan; genius0061@yahoo.com (H.S.A.); shahab.butt01@gmail.com (H.S.A.); sssharif_84@hotmail.com (S.S.); 2 Department of Electrical Engineering, College of Engineering, Majmaah University, Majmaah 11952, Saudi Arabia; 3 Centre for High Energy Physics, University of the Punjab, Lahore 54000, Pakistan; Wasim.naeem123@gmail.com; 4 Department of Physics, Beijing Normal University, Haidian District, Beijing 100875, China; ghulamfarid49@yahoo.com; 5 Department of Physics College of Science, Majmaah University, P.O. Box 1712, Al-Zulfi 11932, Saudi Arabia; m.ashiq@mu.edu.sa; 6 Department of Physics, Forman Christian College (A Chartered University), Lahore 54600, Pakistan; ammaraanwar@fccollege.edu.pk

**Keywords:** ZnO nanoparticles, surface morphology, XANES, optical properties

## Abstract

In this work, microemulsion method has been followed to synthesize vanadium-doped Zn_1−x_V_x_O (with x = 0.0, 0.02, 0.04, 0.06, 0.08, and 0.10) nanoparticles. The prepared samples are characterized by several techniques to investigate the structural, morphology, electronic, functional bonding, and optical properties. X-ray diffractometer (XRD) analysis confirms the wurtzite phase of the undoped and V-doped ZnO nanoparticles. Variation in the lattice parameters ensures the incorporation of vanadium in the lattice of ZnO. Scanning electron microscopy (SEM) shows that by increasing contents of V ions, the average particle size increases gradually. X-ray Absorption Near Edge Spectroscopy (XANES) at the V L_3,2_ edge, oxygen K-edge, and Zn L_3,2_ edge reveals the presence and effect of vanadium contents in the Zn host lattice. Furthermore, the existence of chemical bonding and functional groups are also asserted by attenuated total reflection Fourier transform infrared spectroscopy (ATR-FTIR). UV–Visible analysis shows that by increasing V^+^ contents, a reduction up to 2.92 eV in the energy band gap is observed, which is probably due to an increase in the free electron concentration and change in the lattice parameters.

## 1. Introduction

ZnO is the most prominent and promising semiconductor of the direct band-gap family having a wide band gap of 3.37 eV. It has a potential for numerous device applications such as electronics [1], photonics [2], spintronics [3], biosensors [4,5,6], drug delivery mechanisms [7,8], bioimaging [9,10], and biomedical applications [11,12]. ZnO has been under a wide range of study to understand the root properties such as lattice structure and conductivity, as well as polarity. It has also been engineered using different materials, which show very useful applications of ZnO, but there remain some key obstacles to device development using pure and doped ZnO, particularly the p-type conductivity. For finding the best application of ZnO, it is essential to have a high quality of both n-type and p-type ZnO-based semiconductors, with charge carrier contents of 10^17^ cm^−3^. Basically, ZnO is a diamagnetic in bulk as a single-phase state. However, at room temperature, the magnetic order has been reported in ZnO nanostructures and thin films [13,14,15]. In the last few years, diluted magnetic semiconductors (DMSs) from groups III–V and II–VI attracted very intensive attention on account of their physicochemical properties. These special characteristics are utilized in sensor devices and improve important devices such as spin transistors, logic devices, spin light-emitting diodes (LEDs) [16,17,18], and magnetic memories (MRAM) [19]. It has been predicted that contrary to other DMSs such as GaAs and ZnTe, ZnO in addition to GaN (an important member of wide band-gap family) shows ferromagnetic behaviors at room temperature. In general, the origin of magnetism is mostly due to the incorporation of 3d transition metal ions (such as Mn, Sc, Co, Ni, Cu, Cr, Fe, and Ti) and ions with partially filled f states such as rare earth elements (e.g., Eu, Er, and Gd). These ions are being substituted by the host semiconductor cations. The coordination number of zinc with oxygen, and vanadium atoms with oxygen atoms is one. As a result, the electronic structure of the substituted 3d-transition metal impurities is affected by two main factors: one is strong 3d hybridization among host atoms, and the other is a strong Coulomb force between the 3d–3d subshells of electrons. The multiplet peaks found in the d–d optical absorption spectra are originated from these Coulomb forces. On the other hand, hybridization among the 3d of the dopant and the valance band of the host gives rise to magnetic interaction between carriers in the valence band of the host substance and localized 3d spins. [20]. This fundamental theory about the origin of the magnetic order is under great debate. However, various research groups have investigated transition and rare earth-doped ZnO magnetic semiconductors for wide applications in spintronics and optoelectronics technology [21,22,23,24,25,26,27,28].

It is also evident that contrary to bulk materials, doped ZnO nanoparticles induce some distinct and unusual properties on account of the quantum confinement phenomenon [29]. The nanomaterials’ preparation and doping methods also control for the various properties of doped ZnO [30]. There are many routes to prepare ZnO nanostructures, such as sol–gel, laser ablation, plasma chemical synthesis, the thermal oxidation of metallic zinc, co-precipitation, vapor condensation, hydrothermal synthesis methods, etc. Th different procedures and synthesis conditions control the average size of the particle and their surface morphology [31]. In the current studies, we opted a chemical route to prepare vanadium-doped ZnO powders. This is a low-temperature synthesis technique, which yields a good level of homogeneity in the prepared samples.

## 2. Materials and Experimental Work 

All of the chemicals—zinc acetate [(Zn(CH_3_CO_2_)_2_] (≥99.9 % Sigma-Aldrich, St. Louis, USA), vanadium chloride [VCl_2_] (≥99.9 % Sigma-Aldrich, St. Louis, MO, USA,), cetyl trimethyl ammonium bromide (CTAB) [C_19_H_42_BrN] (≥99.9 % Sigma-Aldrich, St. Louis, MO, USA) as surfactants, and ammonia [NH_3_] (Sigma-Aldrich, St. Louis, MO, USA)—were of analytical grade. Deionized water was obtained from a water deionizer. V-doped Zn_1−x_V_x_O (with x = 0.0, 0.02, 0.04, 0.06, 0.08, and 0.10) compounds were prepared by microemulsion method. The microemulsions were prepared initially by making the stock solution in deionized water of the Zn(CH_3_CO_2_)_2_, VCl_2_, and C_19_H_42_BrN using suitable molar ratios of 0.1:0.1:0.15, respectively. Required volumes for the given composition of V-doped Zn_1−x_V_x_O (with x = 0.0, 0.02, 0.04, 0.06, 0.08, and 0.10) were taken in beakers and then stirring was done at 50–60 °C using a hot plate magnetic stirrer (ISG, Bexwell, UK) with a controlled thermostat (ISG, Bexwell, UK). Meanwhile, aqueous ammonia solution was dripped to sustain a pH value at (~9–10) until the white precipitates were obtained. These precipitates were continuously washed with deionized water to neutralize the solution to pH level (~7). To eliminate any organic-based impurity, methanol was used to wash these precipitates. In the next step, the obtained fine precipitates were dried for six hours at 120 °C in the electric oven (ISG, Bexwell, UK). Furthermore, the calcination was carried out in a temperature-controlled muffle furnace (Nabertherm, Bahnhofstr, Germany) for five hours at 300 °C. After calcination process the materials were ground to fine powders using mortar and pestle and were finally sintered in a furnace at 500 °C for one hour. The prepared powder samples were used to study the structural, surface morphology, functional bonding, and optical parameters. Structural analysis was carried out in an X-ray diffractometer (Model: Philips X-pert-MPD, Malvern Pananalytical Ltd., Worcestershire, UK). The data were analyzed using software such as ‘Unit Cell’ (Department of Earth Sciences, University of Cambridge, UK) and ‘High Expert Pro’ (Malvern Pananalytical Ltd., Worcestershire, UK). To observe the surface morphology of the product, a field emission scanning electron microscope (FESEM) (NOVA Nano SEM200, FEI ThermoFisher, Hillsboro, OR, USA) was used. Functional bonding of the samples was revealed by FTIR (Shimadzu, Kyoto, Japan). An X-ray Absorption Near Edge Spectroscopy (XANES) test at the Zn L-edge, O K-edge, and V L-edge was performed on a Pohang Light Source (PLS-II) using a 10D KIST bending magnet beamline (KIST, Seoul, Korea). All of these calculations were performed at room temperature, and a total electron yield mode was used to record the spectra under 0.01 eV resolutions and a base pressure of 3 × 10^−10^ Torr. Later, the spectra were normalized for steady comparison. The energy band gap was calculated from the reflectance data obtained using diffused reflectance spectroscopy (DRS, Shimadzu, Atsugi, Japan).

## 3. Results and Discussion

### 3.1. Structural Analysis

**Figure 1:**
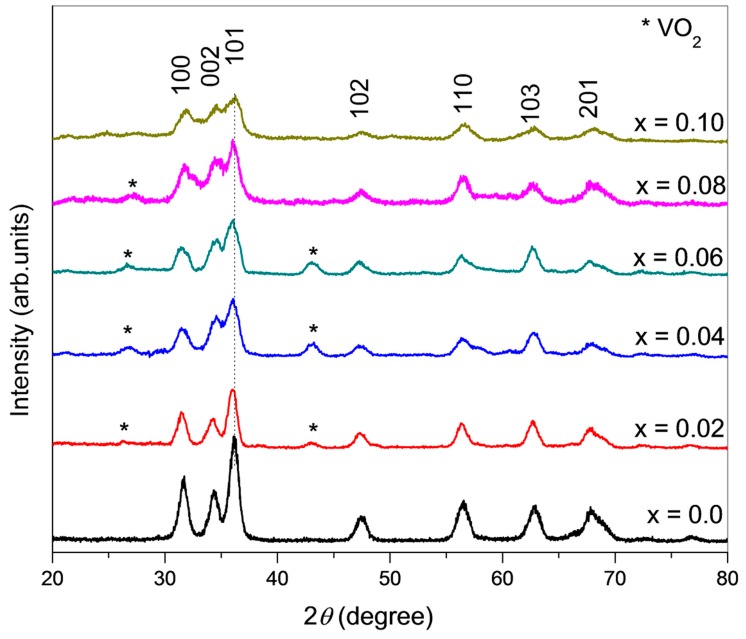
X-ray diffraction spectrum of V-doped ZnO nanoparticles (with x = 0.0, 0.02, 0.04, 0.06, 0.08, and 0.10).

**Figure 2:**
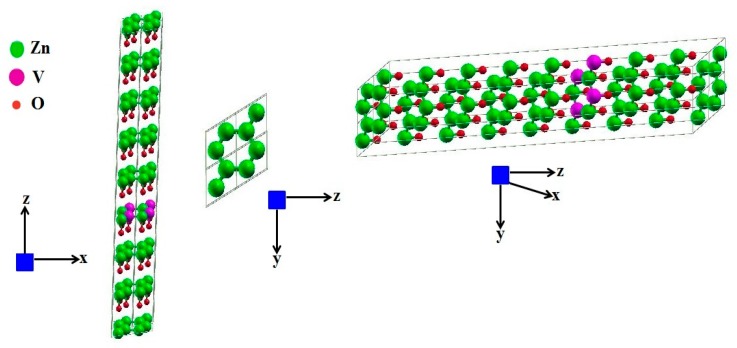
2 × 2 × 2 supercell structure of V-doped ZnO using WIEN2K shown in z-x plane, y-z plane, and 3D (zinc atoms: green, vanadium atoms: purple, oxygen atoms: maroon).

XRD analysis confirmed the crystal structure of pure and V-doped ZnO to be a wurtzite structure. Figure 1 depicts that all the spectral peaks have a good match with the reference data of ZnO (JCPDS card No. 01-089-0510). However, a few peaks at 2θ = 27.345° and 43.01° for x >2% and <8% are identified as a secondary phase of VO_2_ by comparing the results with the JCPDS card No. 00-009-0142. Irrespective of the doping level, the highest intensity peak is of that a certain peak associated with the (101) plane in all the patterns. Lattice parameters such as bond length, crystallite size, and unit cell volume have been calculated from the XRD data using the formula given in the literature [32] and presented in Table 1. It is found that the c-axis elongates with the substitution of vanadium ion, and a gradual left shift in the peak’s position is notable. This shift is evident in an enlarged portion of the XRD spectrum (Figure 1). According to Bragg’s law, it is an established fact that the XRD peaks undergo a left shift under the influence of an increase in the d value. Hence, the proper incorporation of V^2+^ ions on the Zn^2+^ sites is confirmed by this result. Due to the incorporation of vanadium ions, an increase in the c-axis has been observed. The ionic radii of V^2+^ are 0.79 Å, which is slightly larger than the ionic radii of Zn^2+^ (0.74 Å) [33]. It is also confirmed from the ZnO wurtzite supercell structure system, as shown in Figure 2, (produce using WIEN2K software (Institute of Materials Chemistry, TU Wien, Vienna, Austria), that the Zn^2+^ tetrahedral sites are being replaced by V^2+^ ions. This confirms the existence of a V^2+^ stabilized oxidation state after the sintering in the open air at 500 °C, which is in agreement with the literature [34,35]. As the doping proportion is raised, a monotonic decline in XRD peak intensity is observed, which may be accredited to the slight diminution of the crystallinity of the doped product. It is also found that with an increase in dopant concentration up to ~6 %, the lattice constant value increases linearly, which concludes that ZnO has incorporated V^2+^ within its lattice well. However, at higher compositions, x = 0.08 and 0.10, a decreasing trend has been observed, which shows the reluctance in the mixing of V ions. At a higher concentration of vanadium ions, the peaks’ intensity is suppressed remarkably, which also caused secondary phases to disappear. The Scherrer equation was used to find the crystallite size of the single phase [36]:

(1) $$\tau =\frac{K.\lambda }{\beta \cos\theta }$$

where *K* is referred to as the shape factor (here, *K* = 0.9) (a value of *K* =1 usually represents the maximum symmetry in a lattice structure or an ideal crystal shape, such as a perfect cube or sphere), *λ* is the X-ray’s wavelength, and the line broadening at half maximum intensity is denoted by *β*, and *θ* is called the Bragg’s angle. Table 1 shows that with increasing dopant concentration, the average crystallite size decreases. It implies that the doping of V^2+^ can strongly suppress the grain growth of wurtzite ZnO, and hence the decrease in the diffraction peaks’ intensity was noted. Other research works have also aptly justified this observation. [37,38].

These relations are used to find the unit cell volume (v), crystallite volume (*V*), and unit cells per crystallite ($N_{u}$).

(2) $$\left(unit\;cell\;Volume\right)\;v=0.866\;\times \;a^{2}\times c$$

(3) $$\left(crystallite\;volume\right)\;V=\;\tau^{3}$$

(4) Nu=Vv

(5) $$Bond\;length\;\left(l\right)=\;\sqrt{\left(\frac{a^{2}}{3}+{\left(\frac{1}{2}-\mu \right)}^{2}c^{2}\right)}$$

where, $\mu =\;\frac{a^{2}}{3c^{2}}+0.25$.

As a collection of a number of periodically arranged unit cells makes a crystallite, with the increasing dopant concentration, the gradual reduction in crystallite size (shown in Table 1) suggests that the introduction of V ions into the ZnO lattice hinders the possible grouping size to make a crystallite. 

The Rietveld refinements results for the V-doped ZnO have been tabulated in Table 2. The hexagonal structure is also explained by the results of Rietveld analysis, as shown in Figure 2. In general, with the increase of the weight fraction of V, the weighted profile R-factors (Rwp), the profile R-factor (Rp) and goodness of fit (GOF) show a decreasing trend, which implies the accurate pattern fitting [37,38]. The ZnO Rietveld fit phase was performed on the hexagonal wurtzite crystal structure with space group P63mc (186). In this structure, four cations at the corners of the said tetrahedron surround an anion. The unit cell has two units of ZnO, each containing two atoms. For calculating accurate unit-cell parameters, Rietveld refinement is the method of choice. The refinements of the lattice parameters of these phases are a = b = 3.251–3.252 Å and c = 5.21–5.22 Å for all of the samples, while the calculated lattice parameters of ZnO are slightly higher than the bulk value with respect to the expected ones.

### 3.2. X-ray Absorption Near Edge Spectroscopy (XANES)

**Figure 3:**
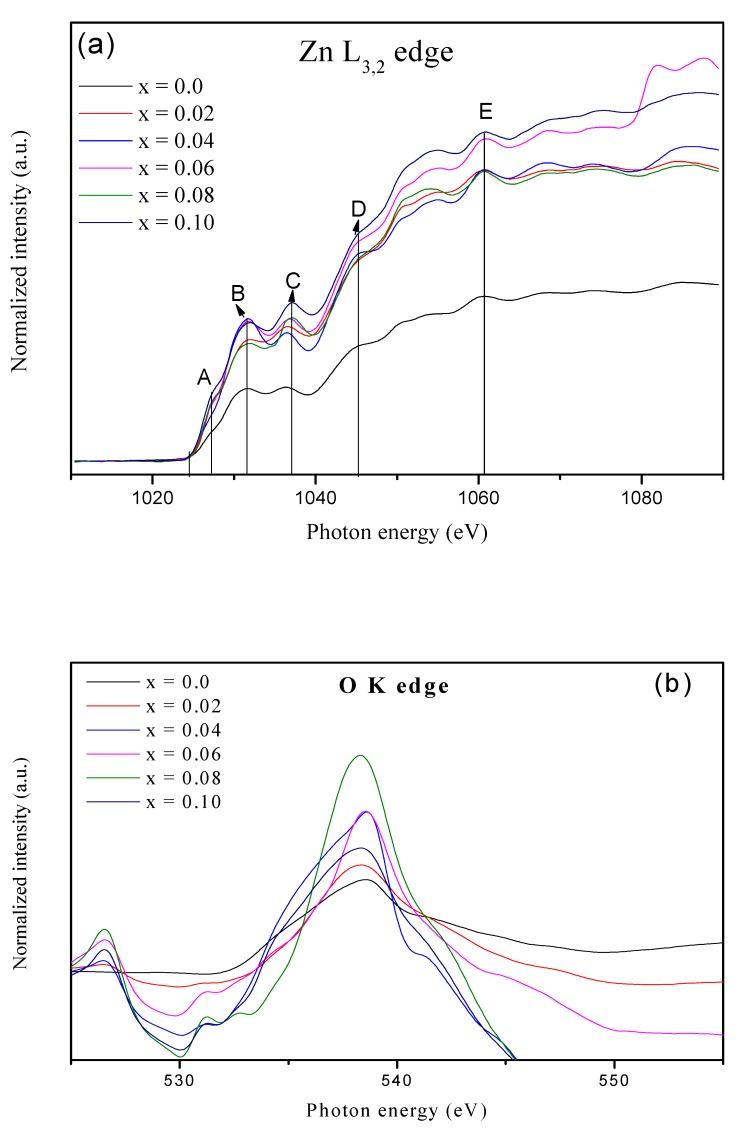

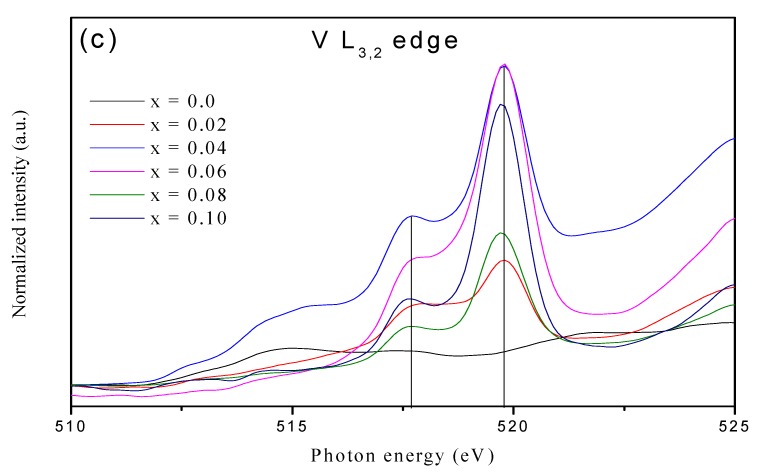
X-ray Absorption Near Edge Spectroscopy (XANES) spectra of (**a**) Zn L_3,2_-edges at different concentration of V contents, (**b**) O K-edge for different concentration of V contents, (**c**) V L_3,2_-edge for different concentration of V contents.

Firstly, a fundamental investigation of the Zn having edge L_3,2_ was executed to study the change caused by vanadium dopants in the average Zn local environment. The L-edge spectra of Zn have been displayed in Figure 3a, which shows the transitions of electrons from 2p_1/2_(L_2_) and 2p_3/2_(L_3_) to the 3d unoccupied state. The energy peaks are not much affected by the presence of V doping, which indicated that the valance shell of Zn remained in the divalent state for all the synthesized samples. In Figure 3a, on the absorption spectra of Zn, five maxima are observed, which are named as A, B, C, D, and E. As the spectrum rises at 1024.5 eV, the energies for the observed peaks are 1027.24 eV, 1031.59 eV, 1036.99 eV, 1045.32 eV, and 1061.30 eV respectively. The pre-edge peak “A” is a result of firmly overlapping wave functions and the 4p–4d levels mixing; hence, it is responsible for its crystal structure, as it provides more convenience to construct larger molecular orbitals. The pre-edge formation is referred to as a t_2g_ low energy level, and it is formed due to the overlapping of Zn 4d and O 2p energy levels. In Figure 3a, the main edge peaks at 1031.59 eV and 1036.99 eV are labelled as ‘B’ and ‘C’ respectively, and they reflect the transition from 2p to 4p energy levels.

For a broader study perspective, the XANES spectra of the K-edge of oxygen (O) is presented in Figure 3b. The high sensitivity of oxygen to the symmetry and local bonding has a key role in oxides because it is connected to all the other constituents of the materials. The O K-edge of ZnO has a wide XANES spectrum as a result of strong overlapping with the neighboring atoms. The O K-edges of the x = 0.02. 0.04, 0.06, 0.08, and 0.10 samples have pre-edges with one peak at 526.6 eV. At x = 0.0, there is no pre-edge peak, However, the samples x = 0.02. 0.04, 0.06, 0.08, and 0.10 all have a “pre-edge” peak, which reflects the endowment of V to bonding. In the O XANES spectra, the pre-edge is an outcome of the forbidden electronic transition due to the atomic selection rules in the electric dipole approximation [39,40]. These pre-edge peaks occur due to hybridization between the oxygen and Zn energy levels. For the O K-edge, the main edge peak appeared at 538.30 eV, which is due to the transition of electrons from the core shell of oxygen 1s to the O 2p.

Similarly, Figure 3c depicts the XANES L_3,2_-edge spectra of the V (d^1^) atom. The pre-edge peak has been identified at 517.676 eV, while the main absorption edge arises at 519.785 eV for x > 0.0. No peak has been observed at x = 0.0, which confirms the incorporation of V^4+^ ions only at x > 0.0. The main absorptions peak appears due to the allowed dipole transition of electrons from $2p^{6}3d^{1}\to 2p^{5}3d^{2}$, which are shifted left by roughly 0.1 eV of lower energy compared to the x = 0.02 spectrum. This is consistent with the replacement of more V^4+^ ions, and also confirms the presence of a V^4+^ stabilized oxidation state. These results are in good agreement with the XRD results, as discussed in Section 1. The main peak L_3_ ($2p_{3/2}$) edge is due to the core hole spin-orbit coupling. The L_2_ edge is relatively broad in broadening from Coster–Kronig Auger decay processes [41,42]. This is to notify that the position of the L_3_ edge and other sharp transitions can be sensitive to the vanadium doping concentration, changes in the oxidation state, and local geometry [43].

### 3.3. SEM Analysis

**Figure 4:**
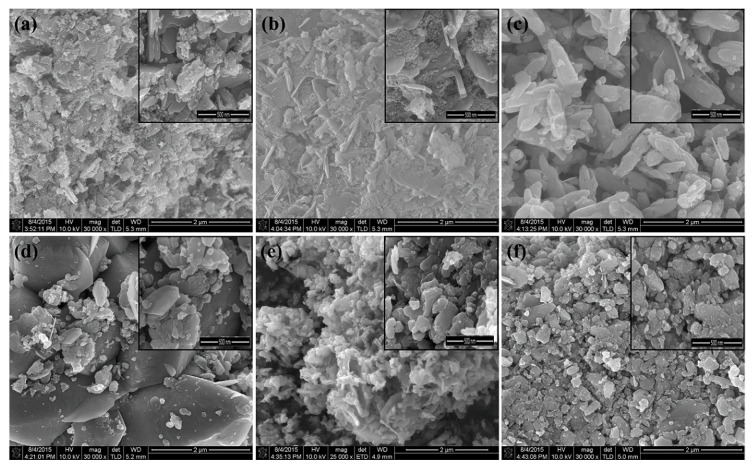
Surface morphology of V-doped ZnO. (**a**) Pure ZnO, (**b**) Zn_0.98_V_0.02_O, (**c**) Zn_0.96_V_0.04_O, (**d**) Zn_0.94_V_0.06_O, (**e**) Zn_0.92_V_0.08_O, and (**f**) Zn_0.90_V_0.10_O.

To study the morphological study of the samples, SEM is considered to be a promising technique. The growth mechanism, sizes, and shapes of the nanoparticles are obtained by SEM analysis. The surface morphology of Zn_1−x_V_x_O (with x = 0.0, 0.02, 0.04, 0.06, 0.08, and 0.10) nanoparticles is presented in Figure 4. These pictures substantiate the approximately spherical shape of the nanoparticles. From the pictures, it is clearly observed that the average particle size was in the range of nanometers. The crystallite size is calculated by the linear intercept method, and it is approximately equal to 12 nm. The obtained crystallite size is in good agreement with the results obtained by the Debye–Sherrer formula. More than 50 different sizes of small particles are selected from every SEM image to take an average of each particle size. It is observed that initially at V = 2%, the grain size decreases with the increase of V concentration; however, suppression in grain size causes agglomeration. As the increase in the surface area to volume ratio occurs, the attractive force between the nanoparticles increases, and hence particles agglomerate. Figure 4d–f show the surface morphology of the Zn_1−x_V_x_O (with x = 0.04, 0.06, 0.08, 0.10) samples, where an agglomeration of the grains become more vivid, and hence the grain size slightly increased, which is in agreement with the literature [31].

### 3.4. FTIR Analysis 

**Figure 5:**
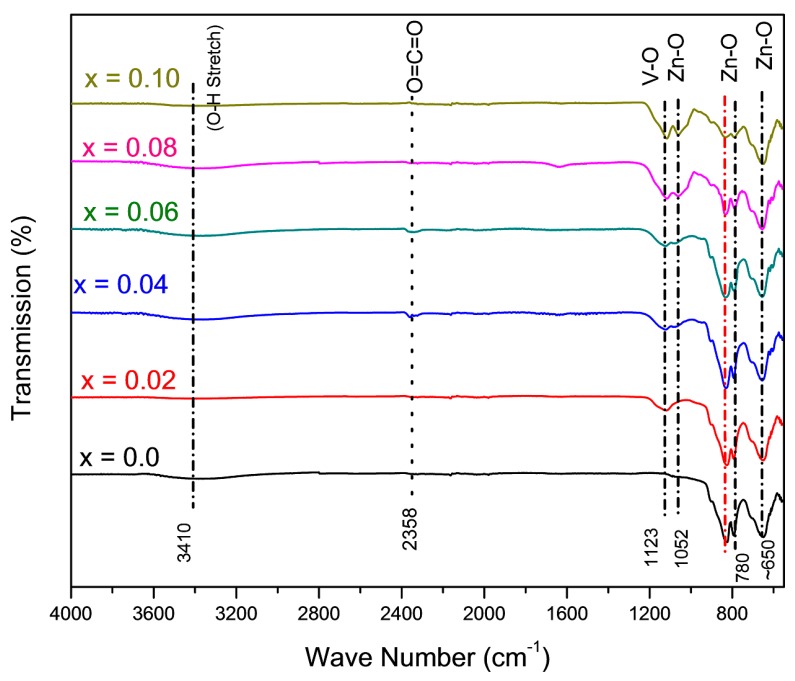
Attenuated total reflection-Fourier transform infrared spectroscopy (ATR-FTIR) spectra of undoped and V-doped ZnO nanoparticles for different V concentrations (i.e., 2–10% vanadium-doped).

Confirmation of the formation of the wurtzite structure of V-doped ZnO nanoparticles has been further studied using FTIR-ATR in 550–4000 cm^−1^ spectral range. Figure 5 depicts the FTIR spectra of pure and V-doped ZnO. The obtained vibration mode at 650 cm^−1^ is related to ZnO and arises due to its stretching vibrational mode [44]. The absorption at 3410 cm^−1^ belongs to the overtone of the O–H stretching mode, which demonstrates the presence of the hydroxyl group [45,46]. Similarly, the absorption at about 1123 cm^−1^ is associated with the V=O band [47]. The absorption peaks between 2300–2400 cm^−^^1^ refer to the CO_2_ molecules in the air [48]. While, the weak absorption peaks that appear at 780 cm^−^^1^ and 650 cm^−^^1^ are related to the vibrational frequencies of vanadium-doped ZnO. These peaks are associated with the change in microstructural features. 

### 3.5. Optical Properties

**Figure 6:**
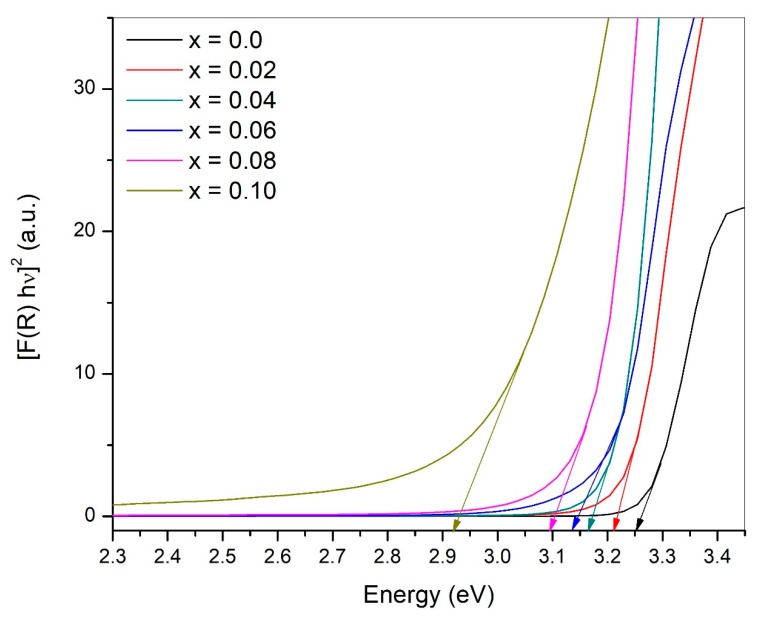
The UV-Visible diffuse reflectance spectra of Zn_1−x_V_x_O nanoparticles with different V ions.

To study the effect of vanadium doping near the optical band-gap region of ZnO, diffuse reflectance measurements were carried out at room temperature. The spectra of Figure 6 display the pure and V-doped ZnO nanoparticles’ behavior in the range of 2.3 eV to 3.4 eV. The energy band gap (E_g_) of Zn_1−x_V_x_O (with x = 0.0, 0.02, 0.04, 0.06, 0.08, and 0.10) nanoparticles have been calculated performing Kubelka–Munk analysis [49] using the following relation:

(6) $$F\left(R\right)=\frac{{\left(1-R\right)}^{2}}{2R}$$

The band gap for pure ZnO is found to be 3.25 eV and for V-doped ZnO (with x = 0.02 to 0.10), it is 3.21 eV, 3.16 eV, 3.14 eV, 3.10 eV, and 2.92 eV respectively. It can be observed that the band gap of V-doped ZnO decreases significantly by increasing the concentration of V in samples. By increasing the concentration of vanadium as a dopant in ZnO enhances the availability of free carriers in the host compound, and thus, a decline in the band gap takes place. However, a few other factors also cannot be neglected, such as the structural parameters (decrease in crystallite size), and the presence of defects because of oxygen vacancies. These factors may lead to the Burstein–Moss shift [50,51]. The Burstein–Moss effect is the process through which the absorption edge is pushed to higher energies, and states near the conduction band become more populated, so the apparent band gap of a semiconductor is increased. According to this effect, more electrons of ZnV_x_O contribute to the conduction band due to the merging of the Fermi level into the conduction band. 

## 4. Conclusions

V-doped ZnO nanocrystalline samples were successfully synthesized through the microemulsion method. The variation in the lattice parameters, such as the c-axis, unit cell volume, bond length, and crystallite size confirm the incorporation of vanadium ions into the ZnO lattice sites. Surface morphology reveals spherical, well-defined nanoparticles. The size of the particles is in agreement with the crystallite size obtained by the Sherrer equation. The existence of a functional group and the chemical bonding of the given samples are analyzed by using FTIR analysis. XANES analysis endorses the presence of Zn, O, and V ions contents in all the synthesized samples. The results reveal that a slight variation has been observed with the substitution of V ions; however, the oxidation states of all the elements in the prepared samples remain unchanged. The UV-Vis results revealed that the band gap decreases by up to 2.92 eV with the incorporation of V contents. From the analysis, it is concluded that vanadium-doped ZnO nanoparticles are the most favorable and promising candidate to be used in applications such as optoelectronic devices.

## Figures and Tables

**Table 1:** XRD parameters for pure and 2–10% vanadium-doped ZnO nanocrystalline materials.

Samples	Pure ZnO	2% V Doped	4% V Doped	6% V Doped	8% V Doped	10% V Doped
*a (Å)*	3.257	3.277	3.283	3.291	3.25	3.24
*c(Å)*	5.20	5.23	5.24	5.24	5.20	5.19
*c/a*	1.60	1.60	1.60	1.59	1.60	1.60
*Cell volume (Å)^3^*	47.8	48.6	48.9	49.1	47.6	47.2
*Bond length*	2.603	2.615	2.620	2.673	2.6	2.56
*Crystallite Size (nm)*	22	19	15	11	19	20
*Volume of crystallite*	484	324	225	121	361	400
*Number of unit cell*	10	7	5	2	7	8

**Table 2:** Refined structure parameters for pure and 2–10% vanadium-doped ZnO nanocrystalline.

Sample	*a = b* (Å)	*c* (Å)	Residual Factors	Values
**ZnO(pure)**	3.251	5.21	R expected Rp Rwp GOF	13.27 30.51 41.196 9.62
**ZnO 2%**	3.254	5.22	R expected Rp Rwp GOF	14.86 17.25 21.72 2.1
**ZnO 4%**	3.251	5.221	R expected Rp Rwp GOF	12.88 13.05 16.05 1.55
**ZnO 6%**	3.253	5.21	R expected Rp Rwp GOF	13.99 18.55 22.80 2.65
**ZnO 8%**	3.248	5.217	R expected Rp Rwp GOF	14.70 21.13 26.7 3.299
**ZnO 10%**	3.252	5.22	R expected Rp Rwp GOF	11.81 17.48 21.69 3.37
